# Airway exposure to multi-walled carbon nanotubes disrupts the female reproductive cycle without affecting pregnancy outcomes in mice

**DOI:** 10.1186/s12989-017-0197-1

**Published:** 2017-05-30

**Authors:** H. K. L. Johansson, J. S. Hansen, B. Elfving, S. P. Lund, Z. O. Kyjovska, S. Loft, K. K. Barfod, P. Jackson, U. Vogel, K. S. Hougaard

**Affiliations:** 1 National Research Centre for the Working Environment, Copenhagen Ø, DK-2100 Denmark; 2 Translational Neuropsychiatry Unit, Department of Clinical Medicine, Aarhus University, Risskov, DK-8240 Denmark; 3 Department of Micro- and Nanotechnology, DTU-Nanotech, Technical University of Denmark, Lyngby, DK-2800 Denmark; 4 Section of Environmental Health, Department of Public Health, University of Copenhagen, Copenhagen K, DK-1014 Denmark; 5 Present Address: Division of Diet, Disease Prevention and Toxicology, National Food Institute, Technical University of Denmark, Søborg, DK-2860 Denmark

**Keywords:** Nanomaterials, Multi-walled carbon nanotubes, Female, Estrous cycle, Ovulation, Fertility, Pregnancy, Developmental toxicity, Reproductive toxicity

## Abstract

**Background:**

The use of multiwalled carbon nanotubes (MWCNT) is increasing due to a growing use in a variety of products across several industries. Thus, occupational exposure is also of increasing concern, particularly since airway exposure to MWCNTs can induce sustained pulmonary acute phase response and inflammation in experimental animals, which may affect female reproduction. This proof-of-principle study therefore aimed to investigate if lung exposure by intratracheal instillation of the MWCNT NM-400 would affect the estrous cycle and reproductive function in female mice.

**Results:**

Estrous cycle regularity was investigated by comparing vaginal smears before and after exposure to 67 μg of NM-400, whereas reproductive function was analyzed by measuring time to delivery of litters after instillation of 2, 18 or 67 μg of NM-400. Compared to normal estrous cycling determined prior to exposure, exposure to MWCNT significantly prolonged the estrous cycle during which exposure took place, but significantly shortened the estrous cycle immediately after the exposed cycle. No consistent effects were seen on time to delivery of litter or other gestational or litter parameters, such as litter size, sex ratio, implantations and implantation loss.

**Conclusion:**

Lung exposure to MWCNT interfered with estrous cycling. Effects caused by MWCNTs depended on the time of exposure: the estrous stage was particularly sensitive to exposure, as animals exposed during this stage showed a higher incidence of irregular cycling after exposure. Our data indicates that MWCNT exposure may interfere with events leading to ovulation.

## Background

Manufactured multiwalled carbon nanotubes (MWCNT) have become attractive commodities for various industries due to inherent properties such as high strength, large surface area, conductivity, and unique electronic properties [1]. Coupled with increased production and application there is however also a significant increase in the risk for occupational exposure, with inhalation being considered the most important route [2].

It has been shown that airway exposure to MWCNTs can induce sustained pulmonary acute phase response and inflammation in the lungs. This is characterized by influx of neutrophilic granulocytes, as well as the production of acute phase protein (Serum Amyloid A, SAA) and cytokines such as IL-1β, IL6 and TNF-α [3, 4], which may lead to systemic inflammation if secreted into the circulation [5–9]. Systemic inflammation can affect an array of tissues and organs with the general agreement that this includes the female reproductive system [10]. The underlying mechanisms involved in systemic inflammation affecting female reproduction are not yet clear, but the hypothalamus seems to be very sensitive to circulating cytokines. For example, in rodents, different immune challenges have been shown to interfere with the luteinizing hormone releasing system at both the hypothalamic and pituitary levels, [11–14]. A series of *in vivo* studies on ewes exposed to endotoxin has shown that inflammation can disrupt the female reproductive axis by for instance disrupting hypothalamic or pituitary signaling leading to impaired reproductive capacity [15–18].

Only a few studies have addressed the potential toxic effects particles can have on female reproduction, be it particles in ambient air or engineered nanomaterials [19, 20] and highlight the importance of continued focus in this area of research. A two-generation mouse study on exposure to ambient air pollution (with a high level of particles) prior to mating showed effects on several parameters pertaining to female reproductive function, including the estrous cycle [21]. To our knowledge, only one study has so far addressed female fertility after MWCNT exposure [22]. Sexually mature female mice were intratracheally instilled with 67 μg MWCNT one day prior to breeding. Time to delivery of litter was significantly delayed, due to a delay in establishment of pregnancy, but no effects were observed for the course of pregnancy or litter parameters [22].

In this study, we have investigated the effects on female reproduction following pulmonary exposure to the MWCNT NM-400. We hypothesized that exposure to MWCNT would induce pulmonary inflammation, which would manifest systemically and thus have the potential to interfere with the female estrous cycle and reproductive function. Estrous cycle regularity was investigated 2 weeks prior to and 2 weeks after exposure to 67 μg of MWCNT. Furthermore, the potential effect on time to delivery of litter was investigated using 2 μg, 18 μg and 67 μg of MWCNT, which are more dose levels than previously used by Hougaard *et al.* [22].

## Methods

### Material and preparation for exposure

The MWCNT NM-400 (Nanocyl-Belgium) was used for exposure. Physico-chemical characterization shows NM-400 to consist of sub-μm long and highly curved MWCNT, with a mean diameter and length of 10 and 295 nm, respectively, and containing approximately 16 wt% of incombustible impurities, dominated by aluminum (5.3 wt%), iron (0.4 wt%) and cobalt (0.2 wt%) [22]. The surface area was 298 m^2^/g [23]. When endotoxin was assessed in the batch of NM-400 by the kinetic Limulus Amebocyte Lysate test (Kinetic-QCL endotoxin kit, Lonza, Walkersville Inc., USA), the concentration was found to be below the detection limit of 0.05 EU/mL [22].

MWCNT were dispersed in vehicle as described in [22], with minor changes. In brief, MWCNT were sonicated for 16 min at 1.34 mg/mL in 0.2 μm filtered, γ-irradiated Nanopure Diamond UV water (Pyrogens: <0.001 EU/mL, total organic carbon: 3.0 ppb) with 2% mouse serum using a 400 W Branson Sonifier S-450D (Branson Ultrasonics Corp., Danbury, CT, USA) mounted with a disruptor horn and operated at 10% amplitude. Samples were continuously cooled on ice during the sonication procedure to prevent excessive sample heating. Lower dose levels were prepared by diluting the stock dispersion with UV water, containing 2% mouse serum, to the desired concentration followed by 2 min of sonication. Mouse serum was prepared in our own laboratory by withdrawal of heart blood from anaesthetized mature female mice (C57BL/6BomTac, Taconic Europe, Ejby, Denmark) into Eppendorf tubes, followed by centrifugation. Serum from several mice was pooled, aliquoted in small vials and kept at −80 °C until use. Serum for control and MWCNT vehicle came from the same vial. Previous measurements showed the concentration of endotoxin to be less than 1 EU/mL of serum or 0.0008 EU/mouse for a single instillation [22]. We have previously assessed the stability of NM-400 in dispersion by DLS, and found it to be satisfactory when the dispersion was vortexed followed by sonication in ultrasound bath for 5 min every 20 min. The apparent particle size number distribution in vehicle was determined by visual inspection and dynamic light scattering (DLS; Zetasizer Nano ZS, Malvern Instruments Ltd., UK) using the Dispersion Technology Software as described previously [22].

### Animals and exposure

The animal experiments complied with EC Directive 86/609/EEC and Danish regulations on experiments with animals (The Danish Ministry of Justice, Animal Experiments Inspectorate, Permit 2010/561-1779 C1). The C57BL/6 J strain is widely used in nanotoxicology, including our previous studies on developmental nanotoxicology (e.g. [22, 24–26]), and was therefore chosen as the test species. In both Experiment 1 and Experiment 2, control and exposed animals were handled in the same manner to avoid differences due to handling.

### Experiment 1: estrous cycle

Female (*n* = 50) and male (*n* = 5) C57BL/6JBomTac mice were delivered to the National Research Centre for the Working Environment by Taconic Europe, Ejby, Denmark, at 9 and 10 weeks of age, respectively. Females were randomly distributed into ten polypropylene cages holding five mice each, and males were housed together in one cage. Animals were housed in an open cage system under controlled environmental conditions (light 6 a.m. to 6 p.m., temperature 21 °C ± 2 °C and humidity 50% ± 5%) with aspen bedding (Tapvei, Estonia), enrichment (mouse house (80-ACRE011, Techniplast, Italy), small aspen blocks (Tapvei, Estonia), nesting material (Enviro Dri, Lillico Biotechnology, UK) and ad libitum access to feed (Altromin 1324, Brogaarden, Denmark) and tap water. Females and males were kept in the same room throughout the experiment and bedding from the male cage was placed in the female cages twice weekly to synchronize estrous cycling (Whitten-effect [27]), until 4 days before initiation of the experiment.

**Fig. 1:**
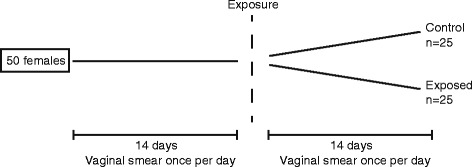
Design of Experiment 1. Vaginal smears were obtained once a day for 14 days from 50 females. Here after, 25 females were randomly chosen for exposure to 67 μg MWCNT by intratracheal instillation, and 25 received vehicle only. Vaginal smears were obtained for 2 weeks more, where after the experiment was finalized and vaginal smears evaluated by a person blinded to exposure status

Three weeks after arrival, and continuing for 4 weeks, females underwent vaginal smearing once daily between 12:00 noon and 01:00 p.m. At day 15 of smearing, five cages with five females (*n* = 5/cage) were selected at random and the females were intratracheally instilled under isoflurane anesthesia (3.5%) [28] with 67 μg of NM-400 in 50 μl of vehicle into the lung lumen followed by 150 μl of air. The control group (five cages, *n* = 5/cage) was sham-instilled with 50 μl of vehicle followed by 150 μl of air. Body weights at time of exposure were 21.0 ± 0.2 (SD) g in controls, and 21.1 ± 0.2 (SD) g in the MWCNT group. On the day of exposure, instillation took place after vaginal smearing. Smearing continued with cages blinded with respect to female exposure status. On day 14 after exposure, the females were killed in Hypnorm–Dormicum anesthesia by withdrawal of heart blood (Fig. 1). Bronchoalveolar BAL fluid was collected from all exposed females (*n* = 25), as well as from ten females randomly selected among the 25 controls, for assessment of cell composition. From eight females in each group, the brains were isolated, and the frontal cortex dissected, snap frozen in liquid nitrogen, and stored at −80° until analysis by quantitative real-time polymerase chain reaction (real-time qPCR). Females were weighed prior to initiation of smearing, before exposure, 4 days after exposure and at termination of study. One female was excluded from the study after 1 week, due to weight loss caused by bad teeth.

### Experiment 2: time to delivery of litter

Naïve C57BL/6JBomTac mice, 100 females and 100 males, were delivered at 8 and 9 weeks of age, respectively and treated as described in [22]. Housing conditions were as those described for Experiment 1, except for change to a specific breeding diet, i.e., Altromin 1314, and provision of only nest material as enrichment during nursing. Upon arrival, animals were randomly assigned to groups of the same sex, and were housed five per cage. As above, males and females were placed together in the animal room and female cages were supplemented with soiled bedding from the male cages. After 1 week, all animals were weighed and males were moved to single housing. Cages with females were semi-randomized, according to weight, into four groups; control (*n* = 30), low (*n* = 20), medium (*n* = 20) and high dose (*n* = 30) and instilled with 0 (vehicle only), 2, 18 or 67 μg of NM-400 in vehicle, respectively. The following day, females were transferred to male cages, one female to one male with cages blinded with respect to exposure status of the female. The females were weighed on a weekly basis and day of delivery registered. Males were removed from the cages when female body weight gain indicated conception or at termination of the cohabitation period of 8 weeks. Impregnated females were allowed to carry their pregnancy through and were checked for birth at least once daily. Time-to-delivery was calculated as the number of days from start of cohabitation to day of delivery (designated postnatal day (PND) 0). The following day (PND 1), offspring was counted, sexed, and weighed, as was the dam.

Additional weights were recorded on PND 8 and 12 (except for offspring of dams that underwent the BAL procedure 4 weeks after exposure). Lung inflammation due to MWCNT exposure was assessed in all adult females by differential cell count of bronchoalveolar fluid, 4, 6 or 8 weeks after exposure (*n* = 8–18/group), as described below. Eight weeks after exposure, the brain was isolated and frontal cortex dissected from 8 to 10 adult females in each group, snap frozen in liquid nitrogen, and stored at −80° until real-time qPCR analysis. The number of implantations was counted in the dams, irrespective of the time point of euthanasia.

### Vaginal smearing and scoring

Vaginal smearing was performed by gently inserting a pipette with 40 μl of 0.9% sterile saline 1–3 mm into the vagina and flushing twice with saline. The liquid was transferred to Eppendorf tubes, briefly shaken and 5 μl were placed on glass slides to dry for up to 30 minutes until fixation in 96% ethanol and staining with May Grunewald-Geimsa. Stained vaginal smears were qualitatively scored using light microscope at 10× or 25× magnification. Depending on the presence of specific cell types, samples were scored as proestrous, estrous, metestrous or diestrous [29]. If more than one cell type was present, the smear was denoted as a transition state. Evaluation of estrous cycles used the estrous state as point of reference; a complete estrous cycle was defined as the time span from the first day of estrous in one cycle to the first day of estrous in the subsequent cycle. The length of all concluded cycles within the study period were noted and used for analyses, apart from one control and one exposed female that were completely excluded from the analysis as they displayed only one cycle each, with durations of 27 and 22 days, respectively. Furthermore, it was noted whether the cycles were regular (with a length of 4–7 days) or irregular (cycles shorter than 4 days, longer than 7 days, with 4–5 consecutive days of estrous or 5–6 consecutive days of diestrous) based on the definitions in [29]. To test if females were more sensitive to the instilled MWCNT at specific estrous stages, females with regular pre-exposure cycles were first categorized according to stage at exposure (i.e., proestrous, estrous, metestrous, or diestrous). For each category, the number of regularly and irregularly cycling females in the post-exposure period was summarized. All data were scored and evaluated for regularity, with the observer blinded to the exposure status of the females. Only when the evaluation was completely finalized, exposure status was revealed.

### BAL preparation and analyses

BAL cell composition and neutrophil granulocyte influx were used to assess lung inflammation after pulmonary exposure to MWCNT. BAL was collected under Hypnorm–Dormicum anesthesia by flushing lungs three times with 1.0 mL of 0.9% sterile saline through the trachea [28]. BAL was immediately stored on ice, until fluid and cells were separated by centrifugation at 4 °C and 400 × g for 10 min. BAL cells were resuspended in 100 μL of medium (HAMs F-12 with 10% of fetal bovine serum and 1% of penicillin and streptomycin). The BAL cell composition of macrophages, neutrophils, lymphocytes, eosinophils and epithelial cells was determined in 40 μL of resuspension, centrifuged at 55 × g for 4 min at room temperature in a Cytocentrifuge 2 (StatSpin), by counting 200 cells fixed and stained as described in [28]. All slides were randomized and blinded before scoring by the same person. The total number of live and dead cells in BAL samples was determined in a further diluted suspension (20 μL of cells in 180 μL of HAM’s F12 medium with FBS and PS) by counting in NucleoCounter (Chemometec, Denmark), following the standard kit procedure. Relative numbers were obtained from the differential cell counts. Unfortunately, differential cell count was not possible for Experiment 1 due to technical errors: cell membranes were destroyed to a degree that did not allow identification and counting of the individual cell types. Qualitative assessment of presence of particles was, however, possible.

### RT- qPCR analysis of brain tissue

*Brain-derived neurotrophic factor* (*Bdnf*)*, Insulin-like growth factor 1* (*Igf-1*)*,* and *Tumor necrosis factor alpha* (*Tnf*_*α*_) were analyzed in frontal cortices. Tissue was homogenized in Lysis buffer (Applied Biosystems, CA) with mixer-mill (Retsch) twice for 1 min (30Hz/s) at 20 min intervals. Total RNA was isolated using the ABI PRISM^TM^ 6100 Nucleic Acid Prepstation (Applied Biosystems, CA) following the manufacturer’s instructions, where 13 mg of homogenized tissue were loaded per well, as previously described [30]. RNA extraction was not successful with tissue from three females in Experiment 2, as the filter was clotted. Aliquots of the RNA solution were taken for both RNA quantification and qualification. RNA integrity and concentration were determined using RNA StdSens microfluidic chips with the Experion Automated Electrophoresis System (BIORAD, CA). RNA purity and concentration were determined using a Nanodrop 1000 Spectrophotometer (Thermo Fischer Scientific). Data on quality, concentration, and purity of the extracted RNA was evaluated with Kruskal-Wallis one-way analysis of variance. Prior to cDNA synthesis, the RNA concentration of the samples was adjusted to match the sample with the lowest concentration determined by the Nanodrop spectrophotometer. cDNA synthesis was performed using random primers and Superscript III Reverse Transcriptase (Invitrogen, CA) following the manufacturer´s instructions, all with a final RNA concentration of 32 ng/μl. The cDNA samples were diluted 1:15 with DEPC water before RT-qPCR cycling. The RT-qPCR reactions were carried out in 96-well PCR-plates using the Mx3005P (Stratagene, USA) and SYBR Green as described previously [31]. The gene expression of eight reference genes (*18sRNA, ActB, CycA, Gapd, Hmbs, Hprt1, Rpl13A, Ywhaz*) and *Bdnf, Igf-1*, and *Tnf*_*α*_ were investigated (primer sequences and accession numbers are given in supplementary material Table S1). Stability comparison of the expression of the eight reference genes was analyzed by the Normfinder software and the best combination selected. Values for each individual were normalized with the geometric mean of the two selected reference genes [32].

### Data analysis

*P*-values < 0.05 were considered statistically significant. Body weights were analyzed by ANOVA, when relevant with repeated measures in days. BAL cell counts were analyzed by one- or two-way ANOVA, in Experiment 2 with dose and week after exposure as factors. Analyses were conducted stepwise, and only if the overall analysis indicated statistical significance of exposure (or interactions including “exposure”) were further analyses undertaken. In Experiment 2, the litter was considered the experimental unit. ANCOVA controlled for litter size in overall and pairwise comparisons, for birth and lactational weights. Gestational parameters were analyzed by Kruskal-Wallis one-way analysis of variance (SYSTAT Software Package 9). Time to delivery of litter was analyzed by the LIFETEST procedure (proc lifetest; SAS, version 9.4).

When analyzing estrous cyclicity in Experiment 1, the differences in cycle lengths between groups were determined using a mixed model (SAS proc mixed). Groups were randomly formed on the day before exposure since slides were not scored and analyzed until experiments were completed. Prior to the exposure, up to two cycles per female were completed and included in the analysis. One female displayed three cycles prior to exposure, the last two cycles were included in the analysis. The cycle in which exposure took place was included as the exposed cycle. For cycles beginning immediately after completion of the exposed cycle, only the length of the first cycle was included in the overall mixed model, as only 5–7 females in each group presented with more than one cycle post-exposure (SAS, version 9.4). In an additional analysis, it was investigated if any particular estrous stage was more sensitive to MWCNT than others. Females were categorized based on estrous stage at exposure (proestrous, estrous, metestrous, or diestrous) and the number of females with regular and irregular post-exposure cycles was summed up for each exposure group. Fisher’s exact test was applied for comparison of regular and irregular cycles in the post-exposure cycle (GraphPad Prism v. 5.0, San Diego California, USA). RT-qPCR data was analyzed by one-way ANOVA, followed by Bonferroni’s multiple comparisons test if a significant difference was found (GraphPad Prism v. 6.00, San Diego, CA).

## Results

### Particle dispersion

NM-400 dispersed well in the vehicle, which was 2% mouse serum in nanopure water. Hydrodynamic number size distribution of the NM-400 dispersions peaked at 33 and 51 nm, in accordance with previous reports (data not shown) [22, 33].

## Experiment 1

### Exposure, lung cell counts and body weights

In Experiment 1, females were monitored for estrous cycling before and after a single intratracheal instillation of MWCNT. At termination of the study (14 days after exposure) BAL fluid was collected by flushing lungs with saline. The mean total cell count in BAL was significantly higher in exposed female mice compared to controls (*p* < 0.0001, supplementary material Figure S1). Differential cell count could not be performed, but qualitative assessment of slides confirmed the presence of black particulate matter in all exposed females, indicating successful instillation. BAL slides from exposed females displayed eosinophilic crystals, corroborating previous findings on MWCNT exposure [34, 35]. For body weights, ANOVA with days as repeated measure indicated no overall statistical significant effect of exposure, but for the repeated measures there was an overall effect of day (*p* < 0.001) and interaction between day and exposure (*p* = 0.015). Separate analysis of each day of weighing did not indicate a significant effect of exposure on any of the days (*p* > 0.2) (data not shown).

### Estrous cycling

**Fig. 2:**
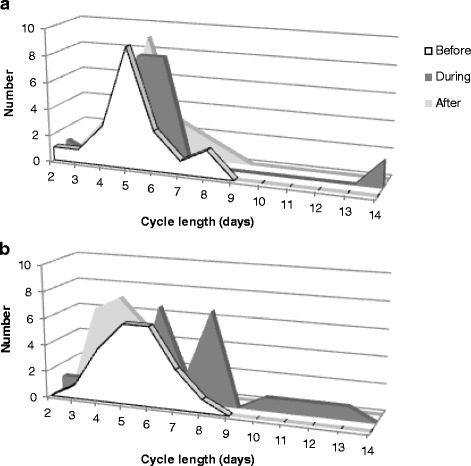
The absolute values for cycle length before, during, and after exposure to 67 μg of MWCNT by instillation for controls (**a**) and exposed (**b**) females are shown (control group *n* = 19–22, exposed group *n* = 21–23)

**Fig. 3:**
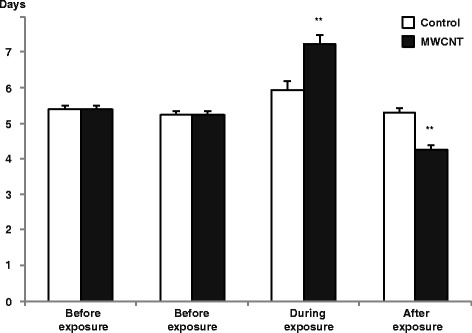
Cycle lengths before, during, and after exposure, obtained from the mixed model SAS analysis. The cycle during exposure was significantly longer than the cycles before exposure. The cycle immediately after exposure was significantly shorter than both the cycles before exposure and the exposed cycle. No effects were observed in the vehicle exposed animals. Values are given as mixed model estimate average ± SEM. (**: *p* = 0.001 compared to the cycle before to exposure; ##: *p* < 0.001 compared to the exposed cycle, *n* = 20–23)

Prior to exposure, the majority of the estrous cycles lasted for 5 to 6 days, as previously described for C57BL mice [36]. Figure 2 depicts cycle lengths immediately prior to, during, and immediately after exposure. Exposure to MWCNT influenced cycle length substantially (Figs. 2 and 3). The mixed model analysis showed a statistically significant effect for cycle (*p* = 0.004) and interaction between cycle and exposure (*p* = 0.022). Exposure to MWCNT increased the cycle length by approximately 2 days, i.e., from 5.3 days before exposure to 7.2 days for exposed cycles (*p* = 0.001). The cycle beginning after exposure was 4.3 days long and thus significantly shorter compared to both the cycle prior to exposure and the exposed cycle (*p* = 0.001 and *p* < 0.0001, respectively). No effects were observed in the vehicle exposed animals (*p* > 0.25). As only a minority of the females (5–7/group) presented with a second full estrous cycle following exposure, this cycle was not included in the overall statistical analysis. The average cycle length in these few females was similar and averaged 5.5 days.

We also investigated if the effect of MWCNT exposure depended on the specific stage of the cycle in which exposure took place, by categorizing the females according to cycle stage at time of exposure. Timing of exposure distributed equally across the different estrous stages for control and exposed females (Table 1). When exposure took place during proestrous and estrous, there were relatively more irregular post-exposure cycles in MWCNT exposed females compared to controls (Table 1, *p* = 0.03). No effect was seen if exposure occurred during diestrous or metestrous. Too few females were exposed during proestrous to allow for statistical analysis of this stage alone.

**Table 1:** Regularity of the post-exposure cycle relative to estrous stage at exposure

		Post-exposure cycle Number of animals with	
Estrous stage at exposure^a^		Regular	Irregular
Diestrous	Control	3	1
	Exposed	1	1
Proestrous	Control	2	0
	Exposed	1	0
Estrous*	Control	5	0
	Exposed	2	5
Metestrous	Control	4	2
	Exposed	6	3

^a^Including only regularly cycling animals during the pre-exposure cycles

*p = 0.027, Fisher's exact test (*p* = 0.026 when proestreous and estrous were pooled)

### Gene expression

No significant effect on gene expression levels of *Bdnf, Igf-1,* and *Tnfα* in frontal cortices of the brain was observed (*Bdnf* 101 ± 6.2%, *Igf1* 99 ± 10%, and *Tnfα* 98 ± 20% (mean ± SEM) compared to control, respectively).

## Experiment 2

In the second experiment, we investigated if exposure to MWCNTs prior to cohabitation affected time to delivery of litter in a dose-related manner. Females were instilled with 0, 2, 18 or 67 μg of NM-400 and started cohabitation with an unexposed male the day after. When female body weight gain indicated conception, the male was removed and female cages monitored for delivery at least once daily. Time to delivery of litter was calculated as the number of days from start of cohabitation to the day of delivery.

### Exposure and cell counts in bronchoalveolar lavage fluid

Total cell count and influx of neutrophil granulocytes in BAL fluid were used as biomarkers of exposure and lung inflammation (Table 2). Numerically, cell counts in the 2 μg dose group were similar to that of the control group, whereas most counts in the 18 and 67 μg dose groups were substantially higher at all time points. The overall statistical analysis showed significant effects of exposure on total cell count, dead cells, absolute and relative numbers of neutrophilic and eosinophilic granulocytes, macrophages, lymphocytes, epithelial cells and eosinophilic granulocytes (only relative number), indicating long lasting inflammation. When the time points were analyzed separately, the effect of MWCNT remained statistically significant for all but eosinophilic granulocytes. Exposure to MWCNT significantly increased the levels of neutrophils in the BAL 4, 6 and 8 weeks after exposure, ≥ 100-fold for the 67 μg dose group. For macrophages and total cell counts similar patterns were observed, albeit with more modest fold changes. Again differences were only statistically significant for the 67 μg dose group.

**Table 2:** BAL fluid cell counts in mice, 4, 6 and 8 weeks post-exposure to 0, 2, 17 or 67 ug of MWCNT NM-400

		Absolute cell numbers				Percentage of cell type in sample (%)			
	Dose level of MWCNT	Control	2 μg	18 μg	67 μg	Control	2 μg	18 μg	67 μg
4 wks	*N*	4	9	9	6	4	9	9	6
	Neutrophils (×10^3^)	7.8 ± 2.9	13.1 ± 7.6	272 ± 168	1173 ± 318^b^	1.1 ± 0.5	1.7 ± 0.9	10.9 ± 3.7	16.2 ± 3.3^a^
	Macrophages (×10^3^)	785 ± 190	607 ± 46	1073 ± 219	4468 ± 662^c^	83.1 ± 2.7	75.7 ± 2.8	76.9 ± 3.7	66.8 ± 4.6
	Eosinophils (×10^3^)	25.2 ± 12	54.5 ± 40	5.7 ± 2.4	106 ± 34	3.9 ± 2.3	4.3 ± 2.8	0.3 ± 0.1	1.4 ± 0.4
	Lymphocytes (×10^3^)	0.7 ± 0.7	2.0 ± 1.5	6.8 ± 3.4	103 ± 52	0.1 ± 0.1	0.2 ± 0.1	0.7 ± 0.3	1.4 ± 0.6
	Total BAL cells (×10^3^)	927 ± 195	821 ± 80	1511 ± 400	6855 ± 1095	-	-	-	-
	Epithelial cells (×10^3^)	109 ± 25	144 ± 11	153 ± 29	1013 ± 230^c^	11.8 ± 2.0	18.2 ± 1.2	11.2 ± 2.2	14.4 ± 1.9
	Dead cells (×10^3^)	237 ± 53	240 ± 23	280 ± 47	943 ± 115	-	-	-	-
6 wks	*N*	10	8	6	10	10	8	6	10
	Neutrophils (×10^3^)	5.9 ± 3.0	5.3 ± 2.2	274 ± 118	2503 ± 792^c^	0.6 ± 0.3	0.8 ± 0.3	9.3 ± 2.7	27.8 ± 5.4^c^
	Macrophages (×10^3^)	802 ± 175	750 ± 123	1867 ± 780	4685 ± 1426^b^	89.2 ± 1.0	94.1 ± 1.1	79.5 ± 3.7	63.2 ± 4.4^c^
	Eosinophils (×10^3^)	18.8 ± 11	1.8 ± 0.7	7.1 ± 57	15.2 ± 13^a^	1.7 ± 0.9	0.3 ± 0.1	0.2 ± 0.1	0.1 ± 0.1
	Lymphocytes (×10^3^)	0.3 ± 0.3	1.4 ± 1.0	26.4 ± 2.5	98.5 ± 46	0.1 ± 0.1	0.2 ± 0.1	1.8 ± 0.6^b^	1.5 ± 0.5^a^
	Total BAL cells (×10^3^)	898 ± 197	793 ± 124	2313 ± 942	7781 ± 2218^b^	-	-	-	-
	Epithelial cells (×10^3^)	71.6 ± 14	34.7 ± 5.4	186 ± 55	479 ± 137^b^	8.6 ± 1.3	4.7 ± 0.8	9.3 ± 1.7^a^	7.4 ± 1.1
	Dead cells (×10^3^)	121 ± 43	121 ± 35	281 ± 131	840 ± 186^c^	-	-	-	-
8 wks	*N*	12	3	3	13	12	3	3	13
	Neutrophils (×10^3^)	10.1 ± 3.8	0.0 ± 0.0	400 ± 169	986 ± 261^b^	1.4 ± 0.5	0.0 ± 0.0	20.3 ± 4.5	22.8 ± 4.2^c^
	Macrophages (×10^3^)	633 ± 89	458 ± 33	1453 ± 678	2346 ± 470^b^	82.7 ± 2.0	85.5 ± 9.0	73.2 ± 2.8	63.8 ± 3.6^c^
	Eosinophils (×10^3^)	2.8 ± 1.6	49.8 ± 48	10.6 ± 5.7	25.6 ± 12	0.4 ± 0.2	8.5 ± 8.3^a^	0.7 ± 0.4	0.8 ± 0.3
	Lymphocytes (×10^3^)	0.7 ± 0.8	1.8 ± 0.9	35.2 ± 31	49.6 ± 1.4^b^	0.1 ± 0.1	0.3 ± 0.2	1.2 ± 0.7	1.5 ± 0.5^a^
	Total BAL cells (×10^3^)	784 ± 130	540 ± 153	2013 ± 945	3830 ± 733^b^	-	-	-	-
	Epithelial cells (×10^3^)	137 ± 40	30.9 ± 5.1	113 ± 8.0	422 ± 108	15.4 ± 1.9	5.7 ± 0.7^a^	4.7 ± 1.5^a^	11.0 ± 1.3
	Dead cells (×10^3^)	174 ± 59	43.7 ± 29	251 ± 63	479 ± 93	-	-	-	-

Mean ± SEM. ^a^, ^b^, ^c^: Statistically significant compared to control mice at the 0.5, 0.01 and 0.001 level, respectively

### Time to delivery of litter

**Fig. 4:**
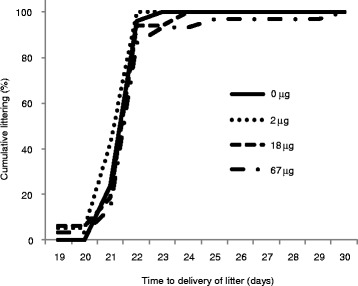
Cumulative littering curves relative to time to delivery of litter. Littering curves were obtained by registration of the day of delivery of the litter relative to start of cohabitation with a mature, unexposed male. Females were exposed to vehicle or 2 μg, 18 μg, or 67 μg of MWCNT by instillation, 1 day prior to cohabitation

During the post-exposure observation period, five females had to be excluded due to technical issues or difficulty in cohabitation with the male, and thus unrelated to MWCNT exposure (three controls: two were found dead shortly after exposure, likely due to accidental damage to the trachea during exposure, and one had been bitten by the male and had to be taken out of the study; and two 18 μg dose females: one found dead shortly after exposure and one had compromised breathing, both likely due to accidental damage of the trachea during exposure). Nine females were not registered for birth, but had implantations at termination of the study (three controls, three 2 μg females, two 18 μg females and one 67 μg female). All remaining females delivered litters. Cumulative littering curves for the exposed females are shown in Fig. 4. A larger proportion of females in the 2 μg dose group gave birth earlier than the control females, whereas females in the 18 μg and 67 μg dose groups delivered with slight delay, compared to controls. Overall, the statistical analysis of time to delivery of litter showed borderline significance of exposure (*p* = 0.0509). Pairwise comparisons showed this to be due to a significant difference in time to delivery of litter between the females in the 2 μg dose group and the 67 μg dose group (*p* = 0.01).

### Birth and lactational parameters

Exposure did not affect gestational nor litter parameters (litter size, implantations, implantation loss, and sex distribution, supplementary material Table S2). For offspring weight during lactation (supplementary material Table S2), one-way ANOVA, with offspring age as repeated measure and litter size as covariate, indicated an interaction between exposure and age (*p* < 0.05). When each day of weighing (postnatal day (PND) 1, 8 and 12) was analyzed separately, there was no significant effect of exposure on PND 1 and 12. On PND 8, analysis indicated an effect of exposure (*p* < 0.05), and pairwise comparisons (litter included as covariate) indicated that this owed to significantly lower offspring weights from females exposed to 18 μg of MWCNT compared to controls (*p* < 0.05).

### Gene expression

**Fig. 5:**
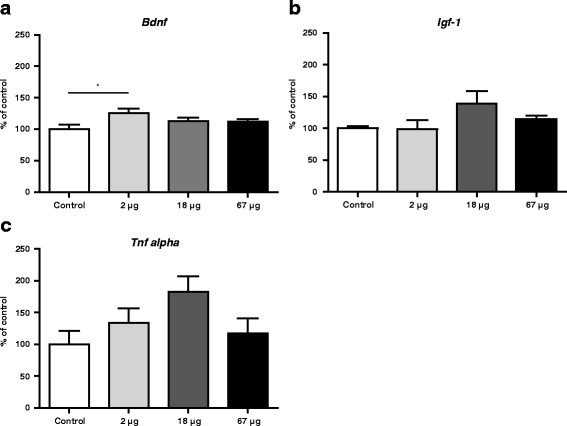
Expression of *Bdnf* (**a**), *Igf-1* (**b**), and *Tnfα* (**c**) 8 weeks after exposure to vehicle or 2 μg, 18 μg, or 67 μg of MWCNT by instillation. Values are given as average ± SEM (*: *p* < 0.05)

In the female brain, *Bdnf* expression was significantly up-regulated following administration of 2 μg of MWCNT when compared to vehicle exposed controls 8 weeks after termination of exposure (F(3,29) = 2.994 *p* = 0.047; Bonferroni's multiple comparisons test *p*˂0.05) (Fig. 5). No other differences were observed.

## Discussion

Two experiments were carried out to investigate if airway exposure to MWCNT can affect female reproduction. The first experiment investigated whether exposure to MWCNT could disrupt estrous cycling, whereas the second experiment assessed if time to delivery of litter, and thus establishment of pregnancy, was affected by MWCNT exposure. It was revealed that MWCNT exposure indeed can influence the length of estrous cycle, but no effects were seen on time to delivery of litter.

### MWCNT exposure and inflammation

Pulmonary exposure to nanomaterials generally results in dose-dependent inflammation as measured by influx of neutrophil granulocytes, both in pregnant and non-pregnant females, concomitantly with increased cytokine levels in lung tissue up to 28 days post exposure [37–39], indicating systemic inflammation. This is especially consistent for MWCNTs, and across a range of MWCNTs with different specific surface areas, lengths and functionalization levels at a dose level of 54 μg/animal [40]. In the present study, MWCNT-exposed females exhibited a five-fold elevated total cell count in BAL fluid compared to controls, strongly indicating inflammation, even if count of neutrophil granulocytes was not available, This is substantiated by findings of the inflammatory properties of the structurally similar MWCNT of NRCWE-026. Twenty-four hours after exposure to 18, 54 and 162 μg NRCWE-026/animal, the lung inflammatory response was characterized by an increase in the total number of cells, predominantly owing to dose-dependent and statistically significant increases in neutrophil granulocyte counts [33]. NRCWE-026 furthermore induced systemic inflammation by dose-dependently increasing plasma levels of the acute phase protein Serum Amyloid A, starting at 54 μg of MWCNT/animal, i.e., a lower dose than in the present study [6]. Furthermore, pulmonary deposition of MWCNT was confirmed by observation of black matter in the BAL fluid from exposed animals. In experiment 2, counts of neutrophil granulocytes was elevated in at all assessed time points alongside total cell counts indicating the presence of lung inflammation during the whole post-exposure period..

### Effect of MWCNT exposure on estrous cycling

Nanomaterial toxicity may arise due to direct action of the material. Studies on bio-distribution of radioactively labeled MWCNT in lungs have shown that up to 7% of the MWCNT translocate to other tissues, primarily local lymph nodes, but also liver and spleen (reviewed in [41]). It is therefore possible that the effects of MWCNT exposure seen on estrous cycling were due to direct effects on the central nervous system (CNS) and/or the ovaries, both of which are involved in the events leading up to ovulation.

However, a more plausible mechanism involves the influence of inflammatory and acute phase responses, which arise in consequence to MWCNT exposure. This immune activation may inhibit reproductive function, especially the tonic secretion of luteinizing hormone (LH) seems sensitive to inhibition as immune stress can delay, or even block, the pre-ovulatory LH surge [42–47]. The effect of acute inflammation on estrous cycling would seem to depend on timing of exposure during the cycle. Battaglia and co-workers have shown that lipopolysaccharide (LPS) affects estrous cycling in ewes when administered during the pre-ovulatory, but not later phases. This is likely due to suppression of the events leading up to the LH surge, i.e., the pulsatile secretion of gonadotropin releasing hormone (GnRH) [47]. In the present study, approximately 60% of the females with 5–6 day long cycles presented with two or more days of cornified cells, the hallmark of the estrous stage. In rats with a 5-day cycle with 2 days of cell cornification (estrous), the LH surge occurred at the end of the first day of cornification, followed by ovulation on the second day of cornification [48]. This rendered these females most sensitive during their first day of estrous and females with 3 days of cornification most sensitive during the second day of estrous. In females with less than 2 days of cornification, the LH surge would occur during pro-estrous. In agreement, we observed significantly more irregular post-exposure cycles in females exposed to MWCNT during the estrous stage compared to control females.

In addition to carbon, NM-400 also contains the metals Al (5.3 wt%), Fe (0.4 wt%) and Co (0.2 wt%), which may have become bioavailable due to sonication of the MWCNT suspension [49–51]. The metals have previously been tested for and associated with effects on female reproduction, however only at high levels of exposure [52–56]. In our study, the females received a single dose of 67 μg MWCNT/animal, corresponding to total amounts of 0.14, 0.011 and 0.005 mg/kg of Al, Fe and Co, respectively. At these dose levels, it is unlikely that the metal impurities would have significantly interfered with female reproductive function to the degree observed in study 1 [56, 57].

### MWCNT exposure and time to delivery of litter

MWCNT did not consistently affect time to delivery of litter. This is in contrast to our previous study, where instillation of 67 μg of MWCNT prior to co-housing with a mature male significantly delayed delivery of litter for an average of 5 days [22]. Our former finding agrees well with the proposed hypothesis that MWCNT exposure induces inflammation that in turn may suppress the female reproductive axis and ovulation. If exposure suppressed ovulation, the females would not attain pregnancy until the subsequent cycle, and a delay of approximately one estrous cycle would be expected. In the present study, however, exposure was not associated with a consistent and significant delay in time to delivery of litter. Furthermore, no effects were observed for the course of pregnancy or litter parameters, which is in agreement with our previous study [22] and developmental studies on gestational airway exposure to other nanosized particles such as carbon black, and titanium dioxide particles [19, 24, 25, 58].

During the pre-mating phase, we kept the naïve females and males in the same room and females were supplied with male bedding to synchronize estrous cycling between females, cf. the Whitten-effect [27]. If the synchronization was successful, it is possible that most females at the day of exposure were in the di- or metestrous rather than the estrous stage, as indicated by Experiment 1 and in [59]. This would potentially leave females less sensitive to cycle disruption by an inflammatory event, which agrees with our observation that times to delivery of litter in exposed and control groups were similar. In light of the findings in Experiment 1, we considered to monitor estrous cycling in Experiment 2, to allow for exposure specifically during the estrous cycle. This would however imply extensive handling of the females that could potentially interfere with cycling and mating. In addition, Experiment 1 did not indicate which day of estrous, if extending across more than 1 day, would be more sensitive. Such extensive changes in study design would also hamper comparison with our prior study. However, in light of our findings, future studies ought to consider monitoring of female cycling prior to exposure.

In humans, a few studies indicate that the particulate fraction in ambient air may impact on female fertility, including time to pregnancy [60–62]. Low grade systemic inflammation arising from exposure to ambient particle level [63] could possibly be a contributing factor. Also, other inflammatory conditions such as obesity and asthma are associated with impairment of reproductive function in women, manifesting as prolonged time to pregnancy [13, 64, 65]. Furthermore, asthma characterized by influx of neutrophilic granulocytes is assumed to foster a more pronounced systemic inflammation, as well as a more pronounced effect on female reproductive function, as compared to eosinophilic asthma [13, 64]. This is of particular interest in light of the present study, as the inflammatory response following lung exposure to particles is also characterized by influx of neutrophil granulocytes [22, 24, 26]. In further support of this notion, a recent meta-analysis of the global gene expression patterns in murine lung following MWCNT exposure showed that the pulmonary transcriptional response to MWCNT was similar to the transcriptional response following bacterial infection models including LPS [66].

### MWCNT exposure and the CNS

Studies investigating molecular changes in the CNS after exposure to CNTs are sparse. Recently, it was reported that release of BDNF may be stimulated in both cortical and hippocampal neurons by CNTs when delivered to primary cultured neurons [67]. Intraperitoneal administration of MWCNT at high dose levels (80 mg/kg and 800 mg/kg) to male mice was, however, associated with an antidepressant-like effect in the forced swim test 2 weeks after administration. In addition, expression of *Bdnf* mRNA, but not protein was changed in whole brain tissues [68]. In the present studies, *Bdnf* expression in the frontal cortex was only significantly upregulated in females 8 weeks after exposure to 2 μg of MWCNT. MWCNT exposure could potentially affect the CNS via the induced inflammation, since administration of pro-inflammatory cytokines and of bacterial lipopolysaccharides has been shown to reduce BDNF in the CNS [69]. Overall, the knowledge of the neurotoxic properties of manufactured nanomaterials remains scarce [70].

## Conclusion

In this study, we have shown that air way exposure via intratracheal instillation to the MWCNT of NM-400 affects estrous cycling in the mouse; the cycle ongoing during exposure was prolonged and the cycle after exposure was shortened. MWCNT was delivered to the lungs via intratracheal instillation, a method delivering the MWCNT as a bolus. This results in a higher dose rate than under realistic inhalation conditions, where delivery of a similar dose may take from hours to weeks. Use of instillation as the means of exposure is therefore not comparable to real-life exposure, but can be used when conducting proof of principle studies and ranking of particle toxicity. Our finding provides foundation to conduct studies involving exposures closer to the real-life scenario and is a step towards bridging the knowledge gap existing between female specific health effects and nanomaterial exposure.
